# Eddy Current Testing of Artificial Defects in 316L Stainless Steel Samples Made by Additive Manufacturing Technology

**DOI:** 10.3390/ma15196783

**Published:** 2022-09-30

**Authors:** Matúš Geľatko, Michal Hatala, František Botko, Radoslav Vandžura, Jiří Hajnyš

**Affiliations:** 1 Faculty of Manufacturing Technologies, Technical University of Košice with Seat in Prešov, 080 01 Prešov, Slovakia; 2 Center of 3D Printing Protolab, Department of Machining, Assembly and Engineering Technology, Faculty of Mechanical Engineering, VSB-TU Ostrava, 17. Listopadu 2172/15, 708 00 Ostrava, Czech Republic

**Keywords:** non-destructive testing, eddy current, additive manufacturing, selective laser melting, stainless steel, artificial defects

## Abstract

Additive manufacturing has many positives, but its incorporation into functional parts production is restricted by the presence of defects. Eddy current testing provides solutions for their identification; however, some methodology and measurement standards for AM (additive manufacturing) products are still missing. The main purpose of the experiment described within this article was to check the ability of eddy current testing to identify AM stainless steel parts and to examine the data obtained by eddy currents variation under the influence of various types of designed artificial defects. Experimental samples were designed and prepared with SLM (selective laser melting) technology. Artificial defects, included in the samples, were detected using the eddy current testing device, taking the important circumstances of this non-destructive method into account. The presented research shows significant potential for eddy current testing to identify defects in AM products, with a resolution of various types and sizes of defects. The obtained data output shows the importance of choosing the right measurement regime, excitation frequency and secondary parameters setup. Besides the eddy current testing conditions, defect properties also play a significant role, such as their shape, size, if they are filled with unmolten powder or if they reach the surface.

## 1. Introduction

Additive manufacturing (AM) is a highly innovative process of parts production, which lies in the sequential fusion of thin material layers and ultimately creates comprehensive three-dimensional products of a desired size and shape. At the early stage of technology, its application was primarily dedicated to the rapid prototyping of models within the development processes, which helped reduce their time. With the increasing development of technology, its application extended to the production of functional parts, even those made of metal materials. The main positive is the possibility of making parts with specific shapes, including various holes and cavities, which would be difficult or even impossible to do with conventional technologies [1]. The SLM (selective laser melting) process represents an innovative version of AM technology, which works with the material in the form of a powder. Additionally, DMLS (direct metal laser sintering) and SLS (selective laser sintering) technologies join this powder bed fusion category according to the ISO/ASTM 52900:2021 international standard. The nature of the individual layers’ creation lies in the transformation of the powder into a solid state, using a high-power laser beam (200–400 W). The transformation of the powder-to-solid state can be run through various mechanisms, such as particle wetting, solid state sintering, liquid state sintering and in the case of SLM, melting. So, within this process, the powder material is heated up to its melting temperature, the powder particles melt and after cooling, a material with a homogenic structure is created. Various parameters influence the SLM process, such as the powder size and density, scanning velocity, laser pulse frequency, part’s surface temperature, laser power, material kind, working speed or parameters related to part creation itself, such as the layer thickness, hatching distance, laser pulse places, step size between pulses and scanning strategy [2,3,4]. Recent research that has focused on the selective laser melting (SLM) process of 316L stainless steel include the evaluation of the microstructure and mechanical properties of the mentioned material after manufacturing by using four different SLM devices [5] as well as the effect of the SLM process parameters, such as hatch spacing on melt pool and as-built quality [6] and laser scanning strategies on texture, mechanical properties and site-specific grain orientation [7]. Additionally, microstructure and mechanical properties were investigated on multi-material bimetallic (316L/CuSn10) material in the study performed by Chen, J, et al. [8]. A different study [9] was devoted to the effect of post-processing operations on the surface characteristics of SLM 316L material. Fu, J, et al. [10] summarized the occurrence of defects in different metal materials produced by additive manufacturing.

Same as in the case of other technologies, certain discrepancies also occur during the additive manufacturing processes, which ultimately influence the final product and its future usability. The previously mentioned parameters necessarily enter the SLM process, which, because of their insufficiently consistent setup, leads to defects in the initiation. They subsequently influence the final component properties, based on which the defects can be categorized into the following groups: geometry and size, surface quality, microstructure and mechanical properties. The necessary type of defects on parts with rounded/curved edges is the staircase effect. With the gradual shortening of the added layers, based on the rounding/slope of the round/curve, the shape of the round/curve is not perfectly smooth, but under a detailed view, it resembles a staircase, in which the size depends mainly on layer thickness [11]. Another common defect is balling, which manifests as spherical particles arising after layer creation. These balls exceed the next layer’s thickness, so they disturb the powder roller motion. The main factors are laser energy density, shielding gas contamination and the powder properties [12]. Additionally, these spherical particles can be subjected to roller influence, which causes the decomposition of the material surface and pits initiation at this area after their detachment [13]. At the high scanning velocity and long distances, the initiation of cracks occurs, which are characterized by their short width and long depth. A combination of high scanning velocity and high laser energy input are crucial for their occurrence. In combination with the tensile residual stresses in the material, a rapid decrease in its lifetime and fracture can arise [11,12]. Porosity can be labeled as an absence of material within its structure, under the influence of a majority of the previously mentioned SLM process parameters, part´s creation parameters, powder properties or even other defects. For this reason, porosity is the most frequently occurring defect in SLM products. Pores can be filled by powder or unfilled by powder [14]. Considering that the material is exposed to the laser energy, which is concentrated into the one small point during the SLM process, the induction of residual stresses is inevitable within the mechanism, which is similar to common laser processes. The key mechanisms are sharp thermal gradients, which occur around the laser influence point. In the final product, tensile stresses are present on top of the component, which gradually change into compressive stresses in deeper layers [15].

The occurrence of the previously mentioned defects significantly influences the application of the AM components in practice. Before introducing remedies for their presence restriction, it is necessary to identify and analyze them and non-destructive testing has promising assumptions for this purpose, which is a highly frequent issue for research nowadays. This fact confirms the rapid increase in publications, mainly in the second decade of the 21st century until now, devoted to this topic. Many studies were focused on the application of X-ray radiation. Zhang, X, et al. [16] used computer tomography for porosity identification in an SLM aluminum alloy (AW 7075) and X-ray diffraction was also used to determine the phase composition of the material. Both technologies were used for similar purposes by Chen, H, et al. [17] but the evaluated material was a composite on an iron base with a carbide additive (Fe2W4C). X-rays were also used as the tool to determine the final fatigue behavior and lifetime prediction of a stainless steel 17-4 PH [18], or in the case of X-ray diffraction, for the microstructure evaluation of a nickel-based alloy (Inconel 625) [19] and as the checking method for a physical analytical model, designated for residual stress identification in metal AM components [20]. Ultrasonic testing was the essence of sensor development by Slotwinski, J A, et al. [21], intended for porosity monitoring in metal AM products. Additionally, laser ultrasonic testing was applied for an in situ evaluation of subsurface defects in AISI 316L stainless steel [22] and for residual stress identification in a titanium alloy (Ti-6Al-4V) [23]. For the detection of microdefects in AM material, an acoustic emission was used by Ito, K, et al. [24].

Since the eddy current testing method, which uses properties of electromagnetism, is primarily intended to be used with the surface and subsurface layers for material identification in a reliable quality, it can be the right choice for identifying some defects in metal AM products. A theory for its use in the field of non-destructive testing was already described in 1976, by Biddle, C [25]. Pulsed eddy currents were used for aircraft riveted structures [26], and one of the first practical applications was for tubes’ defect detection in the nuclear industry [27] and crack depths detection [28]. Besides the classic type defects detection, eddy current testing can be used for conductivity measurements [29], conductive and non-conductive coating thickness evaluations [30,31] and residual stresses identification [32,33]. Additionally, technology provides possible applications within the automation [34].

In recent years, interest in eddy current testing usage for AM products increased. In the research conducted by Du, W, et al. [35], an eddy current testing device was tested for the investigation of subsurface defects and a related parameters effect in a Ti-6Al-AV part, made by additive/subtractive hybrid manufacturing (ASHM), which promises a quality increase in the printed parts. A similar study was also performed on the SLM Inconel 738LC alloy [36]. One of the technologies used during this study, which focused on the evaluation of non-destructive testing for AM parts inspection, was a multi-frequency eddy current method used for conductivity measurements, with the aim of density comparison [37]. Kobayashi, N, with a collective, performed fundamental experiments using the eddy current testing for AM metal products detection [38]. A device, working on the base of the eddy currents, was used for the conductivity measurement within the experiment and was devoted to monitoring the heat treatment influence on DMLS AlSi10Mg specimens [39]. Magnetoresistive sensors were used in the heterodyne eddy current testing of artificial defects in AM samples created by AISI 316L stainless steel [40]. On the same material part made by SLM, several non-destructive techniques, eddy current testing not excluded, were compared for defects identification [41]. For structural health monitoring, the ability of the embedded eddy current testing probes in a laser powder bed fusion (LPBF) product was investigated [42]. Two different designs of the eddy current probes were applied for artificial defects detection in AM samples of stainless steel and titanium [43]. For common defects detection of the LPBF process, active thermography and eddy current testing capabilities were evaluated [44].

This research is focused on the evaluation of eddy current signal variations, under the influence of the artificial defect’s presence of various shapes and sizes in AM stainless steel material samples, designed based on frequently occurring defects in AM products. The lift-off effect and standard penetration depth of the used probes as significant eddy current testing factors are also taken into account, on which the used material conductivity measurement follows. The main aim is to analyze the obtained signal variations and consequently find the appropriate eddy current testing setup for various types of defects in AM products, as well as to act as the preliminary study for follow-up studies in order to create the comprehensive eddy current testing methodology for AM defects identification.

## 2. Materials and Methods

Samples including artificial defects, intended for the research, were designed using Autodesk Inventor 2022 CAD software (San Rafael, CA, USA), taking frequently occurring defects in AM products and measurement device properties into account. The designed samples were transformed into an STL file and were printed in the professional industrial Center of 3D printing Protolab in a Technical University in Ostrava, Czech Republic by the Renishaw (Wotton-under-Edge, UK) AM500E 3D printing system, whose laser (fiber type) power was 500 W, scanning velocity was up to 2000 mm·s^−1^ and possible layer thickness was in the range 20–100 µm. Parts with maximum dimensions, equal to 250 × 250 × 350 mm^3^, could be printed at a velocity of 5–20 cm^3^·h^−1^, based on their properties. The printing technology that this printer used was selective laser melting (SLM), during which the material in the form of powder was fully molten and solidified after the laser impact termination. Within this process, the powder material was heated up to its melting temperature, the powder particles melted and after cooling, a material with a homogenic structure was created. After a certain number of layers were applied in this way, samples of a designed shape were created. The material chosen for this experiment was stainless steel powder with the label SS 316L (see Table 1 for material composition) by the Renishaw company (Wotton-under-Edge, UK) and was printed under precisely defined process parameters in order to reach the best possible structure of the final samples [45].

**Figure 1:**
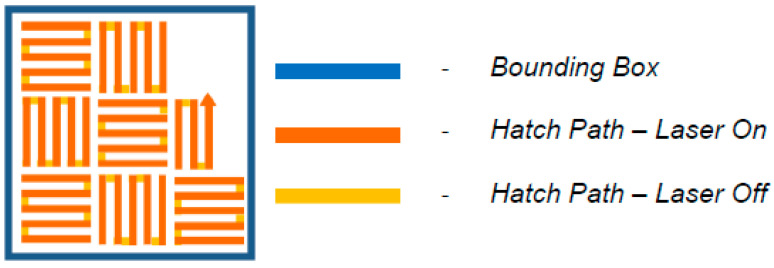
Chessboard printing strategy [45].

The laser power ***P*** for melting the powder was set to 200 W, the scanning velocity ***v*** was 650 mm·s^−1^ and the hatching distance ***d*** was equal to 110 µm. For the layering process, the layer thickness ***t*** was set to 50 µm using the Chessboard hatch fill type (Figure 1), which defines the track of a laser. The laser energy density ***ε*** (energy input) was equal to 55.94 J mm^−3^ according to Equation (1) [12]. The printing parameters were set based on the recommendation of the 3D printing machine and powder material manufacturer, and also on the experience of the 3D printing center (Protolab, Ostrava, Czech Republic) staff. The whole printing system was protected by Argon (Ar) gas during the SLM process.

***ε*** = ***P***/(***v*** × ***d*** × ***t***) (1)

**Figure 2:**
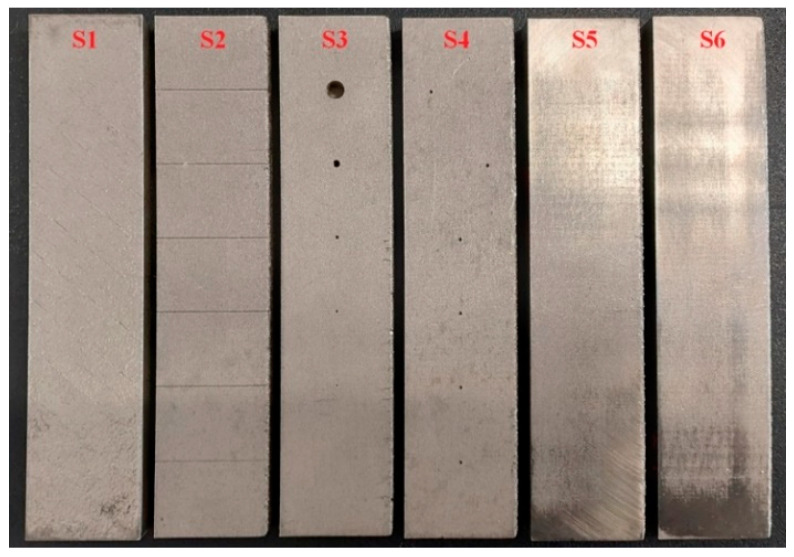
Stainless steel samples made by SLM technology.

Samples (Figure 2), in the number of 6 pieces, were 140 mm long, 30 mm wide and 10 mm thick. As the layer thickness was 50 µm, the samples included 200 layers. However, each of them included various artificial defects that corresponded to frequently occurring defects in the AM processes; namely, thin surface cracks, subsurface pores unfilled by powder and pores filled by unmolten powder [11]. The first sample (S1) was designed for checking the signal variation, during the probing of places with fully molten and partially unmolten powder of various sizes. The second sample (S2), representing surface cracks, was designed with grooves on its surface. The third (S3) and the fourth (S4) samples were designed with 6 blind holes on the bottom surface, with center points situated on a center line in the longitudinal direction. Such holes had a spherical end for representing pores unfilled by powder. The fifth (S5) and the sixth (S6) samples were designed with 6 spherical cavities, with center points situated on a center line in the longitudinal direction, representing pores filled with unmolten powder. All the artificial defects, in all the samples, had a 20 mm distance from the sample edge in the longitudinal direction and between each other. The samples were polished on the side of the testing in order to reach the smoother motion of the probe and decrease the lift-off effect impact, except for the S1 and S2 samples. The reason was that on the S1 sample, the probe was only attached and not moved, and on the S1 sample, the grooves reaching the surface would be blinded by the polishing and the measurement could be limited or even disabled.

**Figure 3:**
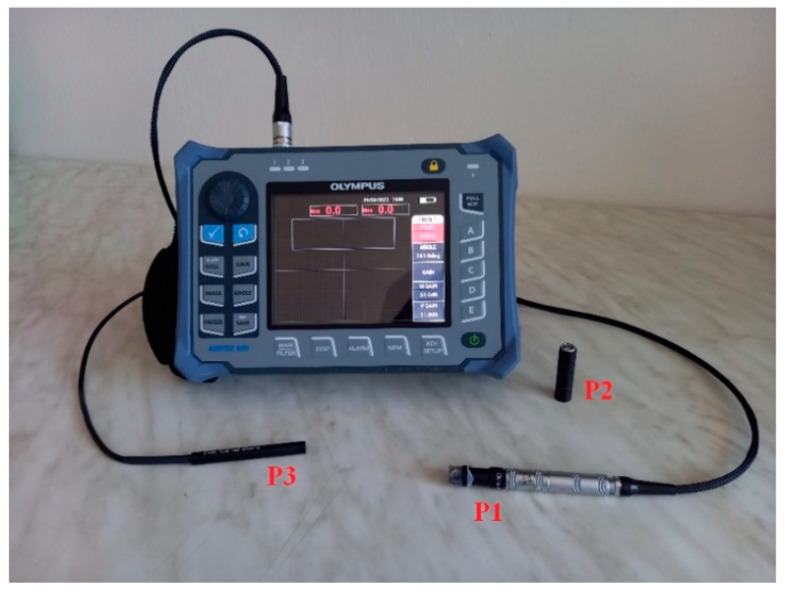
NORTEC 600 eddy current testing device with P1, P2 and P3 probes.

Eddy current testing the of samples was run using the portable NORTEC 600 device (Figure 3) by the OLYMPUS company, Center Valley, PA, USA. The obtained signal of the measurement can be interpreted in 3 regimes. The first is labeled as IMPEDANCE (IMP), in which the obtained curve of a defect was evaluated on a horizontal and vertical axis, relative to the central reference (zero) point. Impedance deviation in a vertical direction (Vmax) refers to its imaginary part (reactance) and a deviation in the horizontal direction (Hmax) represents its real part (resistance). The second is a SWEEP (SWP) regime, in which the voltage variation is represented by a signal deviation over time relative to the central reference horizontal axis, in the form of peaks. The third regime is a combination of the two previously mentioned regimes. The device also provides additional functions and setups, connected to individual regimes. The probes used for the experiment were an absolute (single-coil) type, concretely INDETEC ndt MTW100.S3.A1N (P1), which provides measurements with an excitation frequency in the range of 10–100 kHz, and OLYMPUS S/500 HZ–40 KHZ/0.44 (P2), with a possible excitation frequency between 500 Hz and 40 kHz. For the conductivity measurement, the OLYMPUS SPO-887L probe (P3) was used, for which a 60 kHz excitation frequency is permanently fixed [47]. All experimental measurements were performed at a 21 °C temperature and were set and maintained by air conditioning.

An evaluation of two key parameters preceded the main experimental part. The first was the lift-off effect examination, during which the frequency range and identification ability of the used probes (P1 and P2) were obtained, based on Vmax and Hmax variations under the influence of the probe–sample distance increase at chosen frequencies. The second was the penetration depth calculation, during which the ability of the used probes (P1 and P2) to provide reliable data at a certain frequency was obtained. For the chosen frequency range, including both probes, the penetration depth was calculated and conductivity was also identified as an important factor using the P3 conductivity probe. An artificial defects identification was run specifically for each sample and even for a group of defects within one sample. The first set parameter was the excitation frequency, according to the standard penetration depth and sensitivity. Next, an appropriate regime was chosen and the obtained signal depiction was regulated using additional functions. The parameters’ settings are described and summarized in the results section.

## 3. Results

Before the main experiment, which was dedicated to the artificial defects’ identification, preliminary experiments in the form of a lift-off effect examination and penetration depth calculations were performed.

### 3.1. Lift-Off Effect Examination

**Figure 4:**
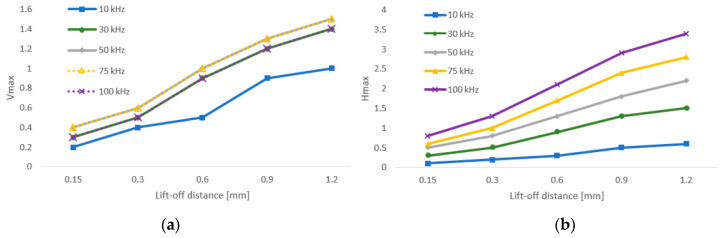
Vmax (**a**) and Hmax (**b**) signal-to-lift-off variation (P1 probe).

**Figure 5:**
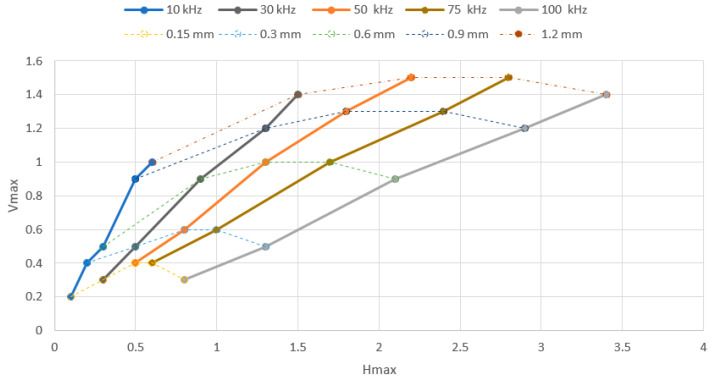
Lift-off effect diagram (P1 probe).

The lift-off effect, which is one of the most important parameters included in eddy current testing, was examined for both of the probes used on the S3 sample on the place without the artificial defect. During its examination, the influence of the probe-sample (lift-off) distance variation on the obtained eddy current signal was evaluated. Concretely, the impedance variation in the vertical (Vmax) and in the horizontal (Hmax) direction was recorded for five chosen frequencies in the frequency range of both probes. A lift-off distance variation was reached by using plastic shims on the probe and sample interface. A total of five thicknesses were chosen, namely 0.15, 0.3, 0.6, 0.9 and 1.2 mm. For the P1 probe, the chosen frequencies were 10, 30, 50, 75 and 100 kHz. The Vmax (reactance) and Hmax (resistance) variations are plotted in Figure 4, and a lift-off effect diagram is plotted in Figure 5. The Vmax variation has an increasing character with the growing of the lift-off distance and also with the elevation of the excitation frequency. As can be seen in the diagram (Figure 4), the growing trend of all the Vmax curves is similar for all, except the 10 kHz curve, which has a slightly different shape. In the case of the 30 kHz and 100 kHz excitation frequencies, the Vmax values were the same, so the curves overlap each other. The same phenomenon occurred in the case of the 50 kHz and 75 kHz excitation frequencies, but at elevated Vmax values. The obtained Hmax variation values were higher in comparison with the Vmax variation values. The values increased with the excitation frequency and also with the lift-off distance. In the case of all five frequencies, the growing trend was the same, which confirms the shape of the curves (Figure 4). By connecting both diagrams, the value of the impedance variation for all five thicknesses were obtained within the lift-off effect diagram (Figure 5), whose shape is typical for lift-off distance examination.

**Figure 6:**
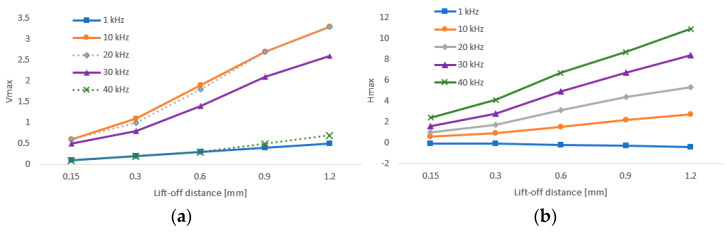
Vmax (**a**) and Hmax (**b**) signal-to-lift-off variation (P2 probe).

**Figure 7:**
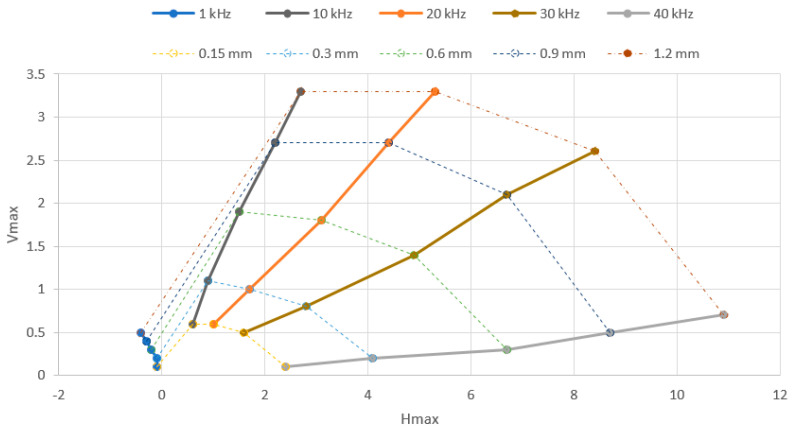
Lift-off effect diagram (P2 probe).

For the P2 probe, the frequencies were set to 1, 10, 20, 30 and 40 kHz. Vmax and Hmax variations are plotted in Figure 6. The P2 probe provided measurements at even smaller frequencies (min. 500 Hz); however, the lowest frequency for the lift-off effect evaluation was set to 1 kHz. The reason was the considerable difference between the lowest and the highest frequency, which can be seen in the P2 probe lift-off diagram (Figure 7). It can be seen that the 1 kHz curve is quite small in comparison with the 40 kHz curve, because the setup parameters were optimal for both boundary frequencies. Another consequence of such a high frequency difference is the maximum obtained Hmax variation at a value of 6.7. This value was reached at 0.9 mm shim for 30 kHz, and at 0.6 mm shim for 40 kHz. So, the values for the 1.2 mm shim at both frequencies and for the 0.9 mm shim at 40 kHz were calculated mathematically by obtaining the right slope of the curve in the lift-off diagram. Similarly, in the case of the P2 probe, the lift-off curve had a typical shape. Additionally, a lift-off effect diagram was obtained in the same way by connecting the separate Vmax (reactance) and Hmax (resistance) variations, plotted in Figure 6. In the case of the Vmax variations, the curves had an increasing trend, by which the three middle frequency curves were similarly shaped and the 10 kHz and 20 kHz curves were partially overlapped. On the other side, both boundary frequency curves (1 kHz and 40 kHz) had a less steep slope and were also partially overlapped. In comparison, the Hmax variation reached elevated values and all the frequency curves had a similar growing trend. Except for the 1 kHz excitation frequency, where all the variations reached negative values, the curves had similar shapes, but with the slope in the opposite direction. Both lift-off effect diagrams also represent the operational range of each probe.

### 3.2. Penetration Depth Calculation

The excitation frequency, which is another important parameter for eddy current identification, was separately set for each sample and even each specific type of artificial defect based on calculations of the standard penetration depth ***δ***, based on Equation (2), in which the eddy currents had a 37% density compared to the density on the surface of the sample. In general, there was a depth limit where the eddy currents were able to identify defects in the acceptable quality [25,34].

***δ*** = √(1/(***π*** × ***f*** × ***µ*** × ***σ***)) (2)

where ***f*** [Hz] refers to the excitation frequency, ***µ*** [H·m^−1^] represents the permeability and ***σ*** [S·m^−1^] is the electrical conductivity. It can be seen that permeability and electrical conductivity are two important material properties that are included in standard penetration depth calculations. Since the 316L is low-carbon austenitic steel with a low magnetic permeability (1.008), it is considered non-magnetic, so this parameter can be excluded from the equation or used in a value equal to one. So, the conductivity is only one of the needed material parameters. The conductivity of the conventionally made 316L stainless steel was equal to 2.33% IACS (International Annealed Copper Standard) [48]. However, considering that materials made by AM processes have different properties, it is more appropriate to use the conductivity of SLM 316L steel. Since the manufacturer does not include information about this parameter in its material sheet or if it is the same for other 316L material sheets from other manufacturers, the conductivity was obtained using the conductivity measurement regime within the NORTEC 600 device and the special conductivity probe (P3). During such a measurement, only the conductivity parameter without the impedance variation was interpreted on the display of the device. The measurement started with the calibration 15 min after turning the device on and connecting the probe because of the accuracy of the obtained data, based on the device manufacturer’s recommendation. During the calibration, the low and the high conductivity limits were set. The low conductivity level was obtained by placing the probe on the calibration block, made from 304 stainless steel, which is also non-magnetic, so permeability (1.020) did not play any role here. Its conductivity is 2.39% IACS and this value was stored as the low limit [49]. The high conductivity level was also obtained by placing the probe on the calibration sample, but in this case, it was made from 7075-T6 aluminum, whose conductivity is 33.48% IACS [50]. This value was stored as the high limit. After the calibration, the probe was placed on the samples made by AM 316L, whose measured conductivity was 3.40% IACS. The accuracy of the conductivity measurement was also checked for the AM material, whose conductivity was listed in the manufacturer´s data sheet. The material was an aluminum alloy AlSi10Mg, with conductivity 21.9 S·m·mm^−2^, which is equal to 37.76% IACS [45]. The measured value of the conductivity when checking the AM AlSi10Mg sample was 34.48% IACS. After the penetration depth calculation for the 10 kHz excitation frequency was performed using the conductivity obtained by the data sheet (37.76% IACS), the penetration depth was found to be 1.08 mm and at the measured conductivity (34.48% IACS) it was 1.13 mm. The standard penetration depths for AM 316L at selected frequencies are listed in Table 2. Additionally, for comparison, the standard penetration depth for conventionally manufactured 316L stainless steel is included. The other parameters, adjusted during the measurement of each sample, were GAIN and ANGLE, by which the first allows the signal to intensify or attenuate, thus improving the quality of the obtained signal on the display of the device, and the second allows the device to rotate the signal over 360° on a display [47].

**Figure 8:**
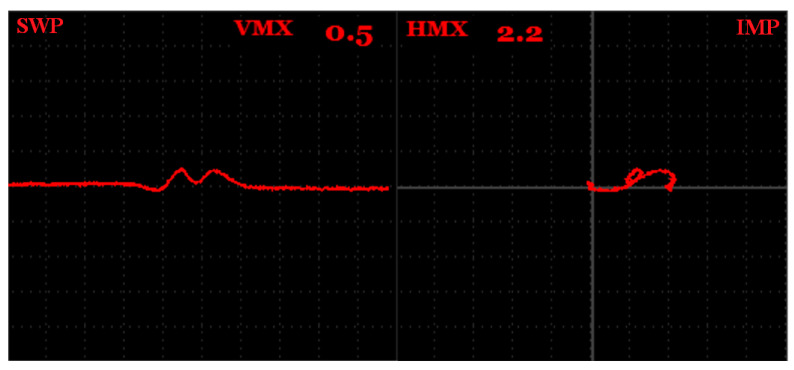
S3 sample, D6 defect signal variation at 60 kHz excitation frequency.

Additionally, the penetration depth was checked on the S3 sample on a R2,5 mm hole (D6 defect). With the appropriate GAIN and ANGLE setup, the signal variation can be obtained even at 60 kHz, where the standard penetration depth is 1.46 mm, based on Table 2. The obtained signal variation is plotted in Figure 8 for the SWEEP and IMPEDANCE mode. The Vmax and Hmax variations reached 0.5 and 2.2, respectively. Taking the shape of the curves into account, for both regimes, both resembled characteristic shapes for such a defect type but in a smaller size. Considering the fact that the mentioned defect is 2 mm below the sample surface, at this frequency it should not have been recorded. Two possible things could have caused this phenomenon. The first is that eddy currents are influenced by this defect bellow the standard penetration depth and such a measurement cannot be considered reliable, despite the trajectory of the impedance curve at the display, which is typical for such a defect. The second reason can be the different material structures, which are manufactured by SLM processes, so they have specific properties that also influence eddy current testing and eddy currents are not attenuated with an increasing penetration depth (skin-effect) to such an extent, such as in the case of conventionally made material.

### 3.3. Artificial Defects Identification

The measurement condition settings are summarized in a Table 3 for each sample and groups of defects, and Table 4 includes the obtained Vmax and Hmax signal variations for the individual defects of each sample.

#### 3.3.1. S1 Sample

The first sample (S1) was designed with four rectangle cavities of a 2 mm depth under the surface, with a 10 mm length and 5 mm width, parallel on a center line in the longitudinal direction. Their thicknesses were 0.2, 0.5, 1 and 5 mm.

**Figure 9:**
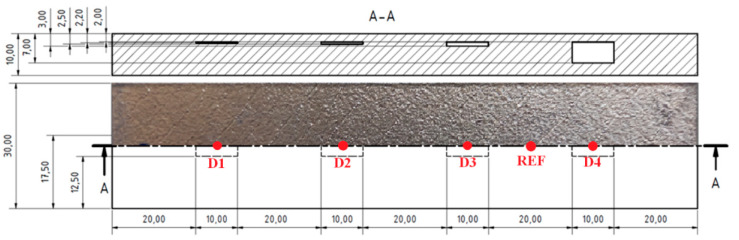
S1 sample.

**Figure 10:**
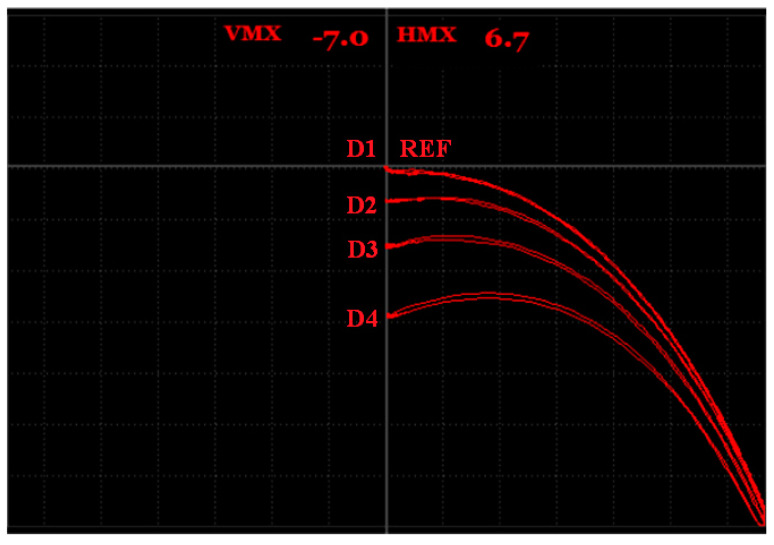
Signal-to-density of unmolten powder variation in S1 sample.

During the measurement, to check the signal variation according to the density of the unmolten powder, an IMPEDANCE regime was used. The frequency was set to 30 kHz for better sensitivity. A middle cross (reference point) was moved to the 70% position on the vertical axis and the air signal (lift off) was rotated over 225° to the right bottom point, using the ANGLE function. Additionally, the GAIN function was used to better distinguishing the obtained signal variations at each place of probing (see Table 3). A measurement itself was performed during the placing of the probe on the first refence (REF) place on the sample with fully molten powder. This signal was set as the reference in the middle of the cross (the zero point). After setting the reference signal, the probe was placed on each of the defects (D1–D4) according to Figure 9. It can be seen that the signal variations increased with the increasing density of the unmolten powder, since the highest deviation value (Vmax = −0.3) was obtained on the D4 defect (Table 4). The signal variation on the D1 defect was only −0.1 of the Vmax, so it was partially overlapped with the reference point. The Vmax and Hmax values, plotted in Figure 10, represent signal variations in the air (lift off) during the changing of the probe position, due to the fact that the device displayed only the maximal obtained signal variation. This introductory measurement was very important for finding out that signal variation occurred if the sample included imperfect structure areas.

#### 3.3.2. S2 Sample

**Figure 11:**
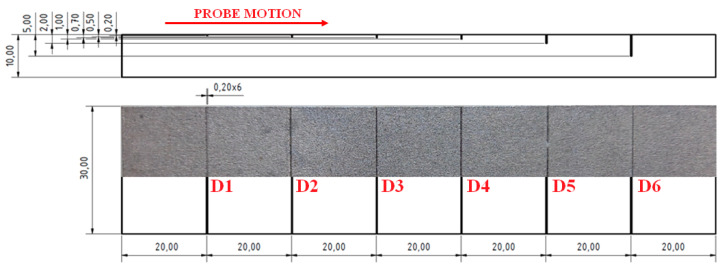
S2 sample.

The second sample (S2) included grooves, and their width was 0.2 mm and their length was equal to the whole width of the sample. Their depths were 0.2, 0.5, 0.7, 1, 2 and 5 mm (Figure 11).

**Figure 12:**
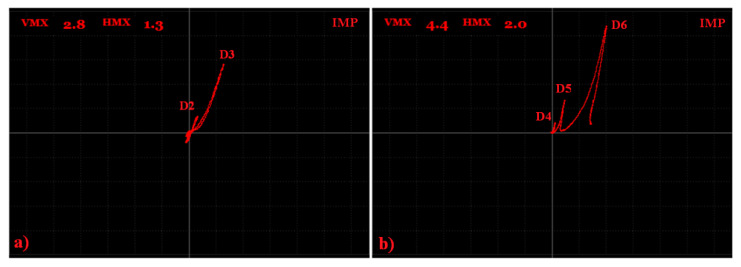
S2 sample, signal-to-size of defect variation; (**a**) D2 and D3 defects at 90 kHz excitation frequency; (**b**) D4-D6 defects at 5 kHz.

As was mentioned before, the S2 sample was not polished, so special tape was used on a probe to prevent the probe wear and measurement quality degradation. Since the big signal variation difference was obtained between the D2 and D6 defects at the same frequency, an inspection was divided into two partial measurements. Both of the measurements were run using the IMPEDANCE regime, but under different conditions. As the first three defects reached a smaller depth, for better sensitivity, a higher frequency (90 kHz) was used. Due to the loss of the signal in the noise signal in the case of the first defect, D1 was excluded of the measurement. At the right ANGLE and GAIN setting (Table 3), clear signal variations were obtained with maximal values of 2.8 at the vertical and 1.3 at the horizontal axis for the D3 defect (Figure 12a). For defects D4–D6, the excitation frequency was set to 5 kHz due to the higher depth of the last D6 defect, by which, consequently, the higher signal deviation was obtained (Vmax = 4.4; Hmax = 2.0), which can be seen in Figure 12b. The signal obtained by all the probed defects had a similar shape in the form of a sharp peak, with a significantly higher deviation in the vertical axis. Such a signal shape can be supposed for thin, surface-reaching cracks, which are one of the frequently occurring defects in AM components. At the right calibration, using the experimental sample as the reference, the depth of such cracks in AM components, made by similar material, can be inspected.

#### 3.3.3. S3 Sample

The third sample (S3) had blind holes with spherical ends of a 2 mm depth on the upper surface of the sample with various radiuses; namely, 0.10, 0.25, 0.35, 0.5, 1 and 2.50 mm.

Since defects in the S3 sample were evaluated on the subsurface layer, a higher attenuation occurred during the IMPEDANCE regime and the signal was quite difficult to analyze, mainly in the case of smaller artificial defects, so the SWEEP regime was selected. The identification was divided into two measurements, concretely one for smaller defects (D1–D3) and one for bigger defects (D4–D6), as the consequence of the fact that the SWEEP regime worked as the signal variation during that time. Additionally, different excitation frequencies were set for both measurements because of their sensitivity.

**Figure 13:**
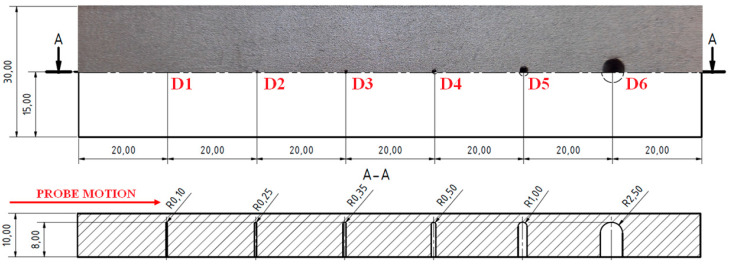
S3 sample.

**Figure 14:**
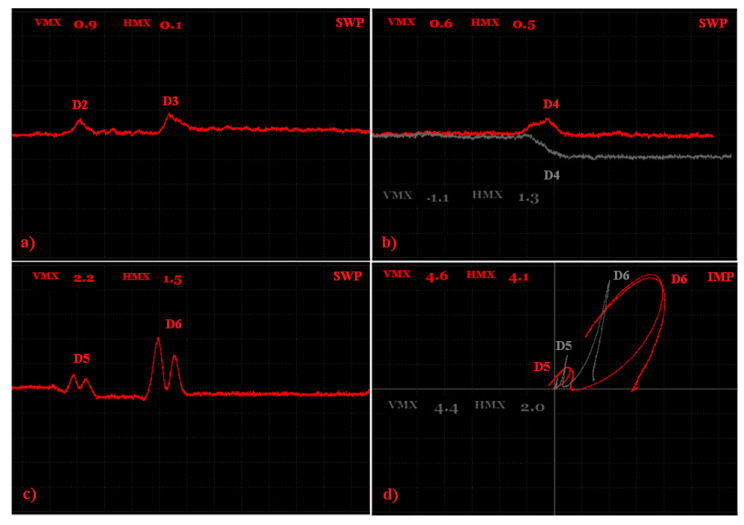
S3 sample, signal-to-size of defect variation; (**a**) D2 and D3 defects at 15 kHz excitation frequency; (**b**) D4 defect at 10 kHz, 135 deg. (grey curve) and 85 deg. (red curve) ANGLE comparison; (**c**) D5 and D6 defects at 10 kHz; (**d**) S3 (reversed) sample, D5, D6 (red curve) defects at 10 kHz and S2 sample, D5, D6 (grey curve) at 5 kHz comparison.

As can be seen in the Figure 13, the D1 defect was not visible with the eye and any signal variation was not recorded, so it is possible that whole defect was not made by SLM technology. Using the 15 kHz frequency, the obtained signal variations by the D2 and D3 defects are plotted in Figure 14a. It can be seen that a higher peak was detected in the case of the D3 defects, with the following values: Vmax = 0.9 and Hmax = 0.1. The highest peak was, of course, received by the D6 defect (Vmax = 2.2; Hmax = 1.5) after using the 10 kHz excitation frequency. Additionally, in Figure 14c, it can be seen that the signal obtained by the D5 and D6 defects was so strong that the typical M shape of the peak for such a defect occurred. Under the same conditions, the D4 defect was also probed, but the slope of the curve on the vertical axis occurred in its case, which can be a little difficult to identify within the measurement of the higher number of defects. So, ANGLE was rotated from 135 to 85° and a better-looking curve with a peak was obtained. The comparison of these two curves and signal variation values are plotted in Figure 14b. Additionally, D5 and D6 defects were probed by the opposite side, where the holes reached the surface of the sample. The obtained signals were compared with the signals of the D5 and D6 defects within the S2 sample. It can be seen (Figure 14d) that the shape of the signal curve had a waving character and, consequently, a higher Hmax variation was obtained than in the case of the S2 sample defects. Such a signal shape is typical for circular defects, reaching the surface of the evaluated material. At the right setting of the calibration conditions, mainly bigger size defects without unmolten powder can be simply detected on the surface and subsurface layers of the material.

#### 3.3.4. S4 Sample

**Figure 15:**
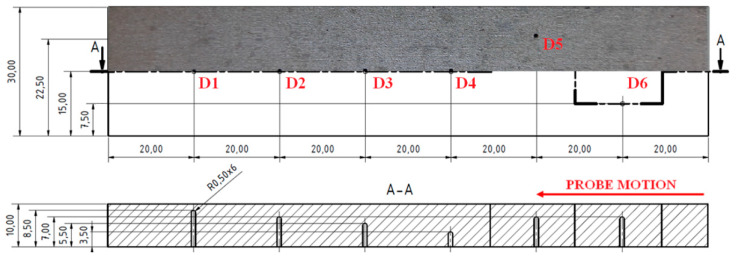
S4 sample.

The S4 sample had blind holes with the same radiuses equal to 0.5 mm, but their depths from the upper surface (real defects positions) varied; namely, 1.5, 3, 4.5, 6.5 mm and another two holes with ends with a 3 mm depth were situated 7.5 mm from the center line in the width direction (Figure 15).

**Figure 16:**
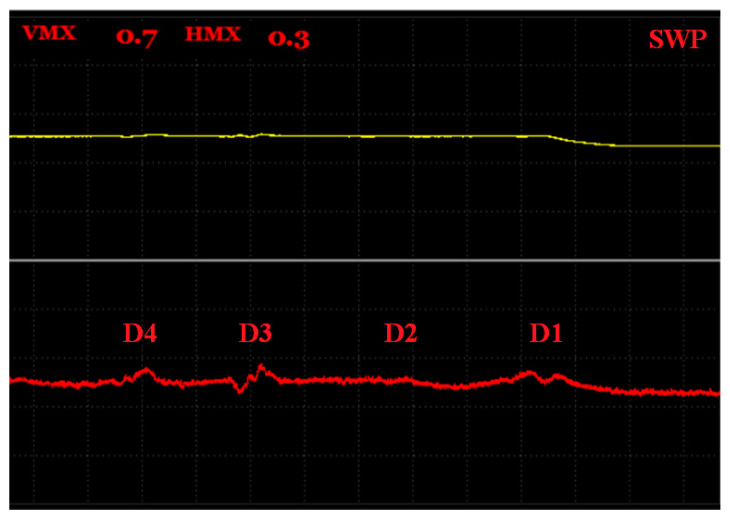
S4 sample, signal-to-depth of defect variation at 15 kHz excitation frequency.

Because the SWEEP regime seems to be better for smaller defects, according to the measurement of the S3 sample, it was also chosen for S4. Additionally, the AUTO XY filtration mode was applicated to better distinguishing and depict the obtained peaks. As the D5 and D6 defects did not cause a significant signal deviation during the probe motion through the center line of the sample, they were excluded from identification and probing started in their position. The excitation frequency was set to 15 kHz to obtain a reliable sensitivity. It can be seen (Figure 16) that the most significant signal variation (Vmax = 0.7; Hmax = 0.3) was detected on a D1 defect, whose position was the closest to the surface of a sample. Additionally, its curve shape resembled the specific shape of such a defect the most. A D4 defect reached a greater depth than the D3 defect, so its peak was consequently smaller. Interestingly, the D2 defect signal was supposed to have values and a peak between D1 and D3, but it somehow had smaller results. The reason can be the imperfect shape of the artificial defect, caused by the SLM technology and the residual powder captured in a hole, which was not removed to a sufficient extent, or maybe a combination of these two factors.

#### 3.3.5. S5 Sample

**Figure 17:**
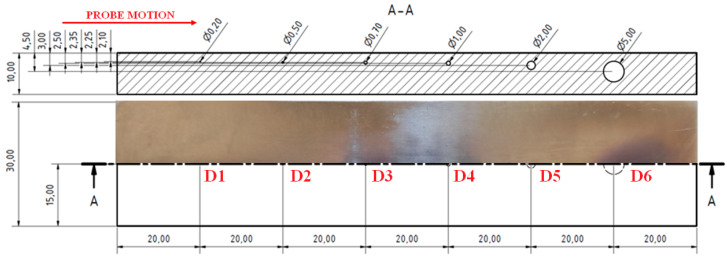
S5 sample.

The fifth sample (S5) had the upper parts of all the inner cavities in a 2 mm depth from the upper surface of the sample, with various diameters; namely, 0.2, 0.5, 0.7, 1, 2 and 5 mm (Figure 17).

**Figure 18:**
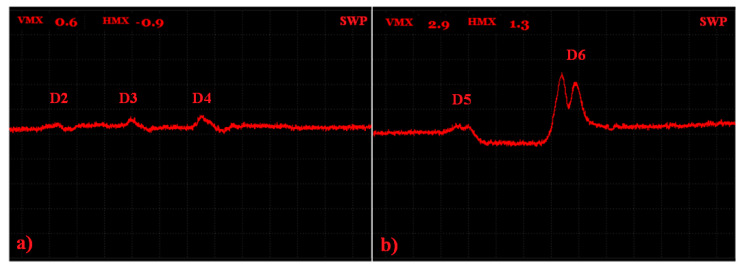
S5 sample, signal-to-size of defect variation; (**a**) D2–D4 defects at 50 kHz excitation frequency; (**b**) D5 and D6 defects at 15 kHz.

As was used in the case of the previous S3 and S4 sample, the SWEEP mode was also chosen for the S5 sample and a detection was separated into two measurements for bigger (D5, D6) and smaller (D2–D4) defects. Same as in the S3 sample, the D1 defect did not cause any signal deviation, so it was excluded from the measurement since it was supposed to be uncreated by the SLM technology. For both measurements (the smaller and bigger defects), the excitation frequency was set to higher values in comparison with the S3 sample. This was because a higher attenuation was caused by the presence of powder in the pores than in the pores without any powder. For the D5 and D6 defects, the frequency was set to 15 kHz, as well as for the smaller defects, even on 50 kHz, which showed reliable obtained data. The shape of the curve in Figure 18a shows gradually increasing peaks with the increasing diameter of the spherical artificial defects. The highest signal variation was detected on a D6 defect with a Vmax value equal to 2.9 and an Hmax value equal to 1.3 (Figure 18b). The shape of the curve was quite similar to that obtained of the D6 defect in the S3 sample (Figure 14c). The D5 signal curve resembled the shape of the combination of D6 and smaller defects and its signal variation value was between them. This can be the proof that smaller defects can be detected by this identification method, but obtained signal variation curves do not have such clear shapes, which is characteristic for these kind of defects.

#### 3.3.6. S6 Sample

**Figure 19:**
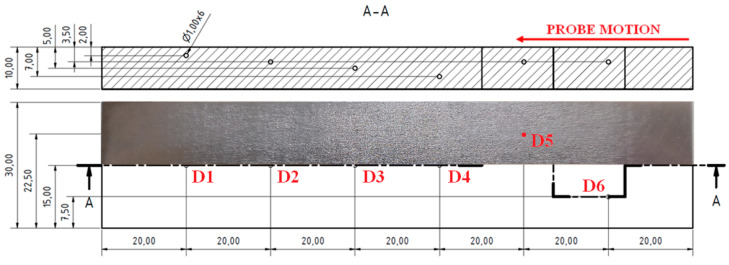
S6 sample.

The S6 sample had cavities with the same diameter equal to 1 mm, but their positions in depth varied; namely, their upper parts had a 1.5, 3, 4.5 and 6.5 mm depth and another two cavities had a 3 mm depth, and were situated 7.5 mm from the center line in the width direction (Figure 19).

**Figure 20:**
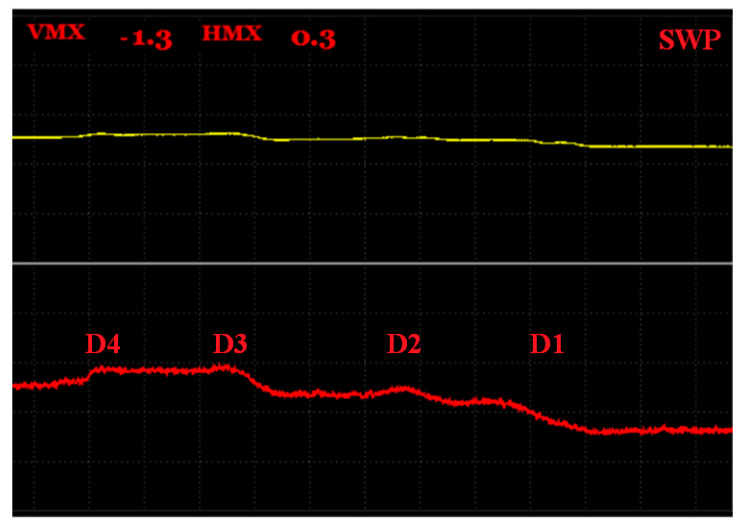
S6 sample, signal-to-depth of defect variation at 25 kHz excitation frequency.

For the last S6 sample, the SWEEP regime was chosen and the AUTO XY mode was set to distinguish and depict the obtained peaks. Similar to the measurement of the S4 sample, D5 and D6 were excluded from detection by the consequence of the same reason. Additionally, the same probe’s starting position and motion direction was selected. The excitation frequency was set to 25 kHz. As depicted in a Figure 20, the obtained peaks were not quite characteristic and significant, as if they were within the measurement of the previous samples. However, numerical values of the signal variations were obtained and the highest refers to the D1 defect, which was closest to the surface, with a Vmax value of −1.3 and an Hmax value of 0.3.

**Figure 21:**
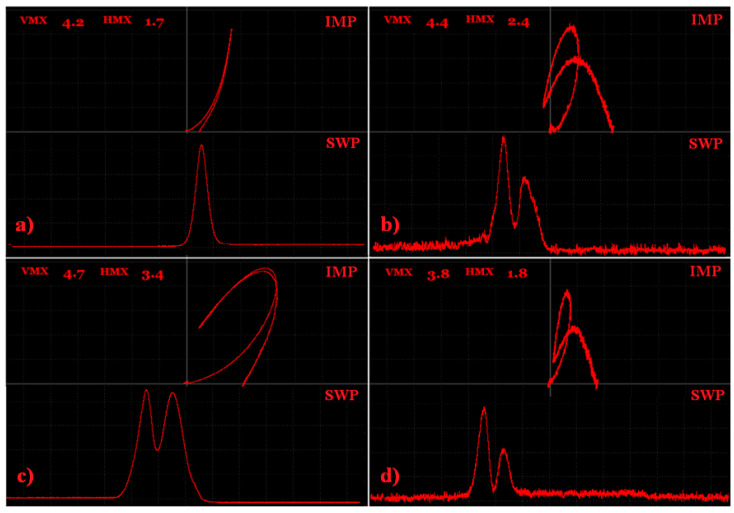
D6 defects signal variations; (**a**) S2 sample; (**b**) S3 sample; (**c**) S3 (reversed) sample; (**d**) S5 sample.

Additionally, D6 defects in the signal variations in the S2, S3, S3 (reversed) and S5 samples at the same frequency (10 kHz) were compared for the SWEEP regime in the same way as the IMPEDANCE regime (Figure 21).

It can be seen that the biggest signal variation was obtained in the case of the blind hole (reversed S3) measured on the side, where it reached the surface (Figure 21c). Concretely, the Vmax was 4.7 and Hmax was 3,4. Smaller values were reached in the case of a groove on sample S2, mainly for the Hmax (Figure 21a). This was because the smaller density of the eddy current was disturbed by the groove in comparison with a hole. Both (S2, reversed S3) of the obtained signals had much smoother curves than S3 (Figure 21b) and S5 (Figure 21d). This was because the defects in the S3 and S5 samples were under the surface and the signal was more attenuated, which caused the noise. Additionally, a higher GAIN was set for these two samples for a better depiction. If the shape of the signal curve is taken into account, for the IMP regime, it can be seen that in the case of the groove, a strong peak mainly in a vertical direction was obtained because of the much higher value of the Vmax than the Hmax variation. In comparison, another three signals of holes and spherical cavities resembled a wave shape. Additionally, the difference between the Vmax and Hmax values was smaller. Here, if the defect reaches the surface, it is important that it is empty or filled by powder. It can be seen that the strongest signal variation was obtained for the defect reaching the surface (Figure 21c) and the weakest was in the case of the spherical cavity filled by powder (Figure 21d). A similar phenomenon occurred in the case of the SWP regime, where in the S1 sample, a strong single peak was obtained and in the case of the reverse S3 sample, a double peak was obtained, which resembled the letter M. Additionally, the same M-shaped peak was obtained in the S3 and S5 samples, but with a stronger attenuation, mainly on the right-side peak and to a greater extent for the D6 defect within the S5 sample.

## 4. Discussion

After the obtained identification data analysis, some similarities with previous experiments conducted by other authors were found. The effect of the lift-off distance on the impedance variation, which was examined within the experiment, was also described as an important parameter in the study conducted by Du W., et al. [35]. Another experiment conducted on Inconel 738LC alloy material showed that the eddy current signal variation increased with the increasing defect size [36], which was found during this research. The lowest detected inner defect was 0.5 mm in diameter, which corresponds with the research performed by Kobayashi N., et al. [38]. As the variation in the signal increased with the crack growth approaching closer to the sensor in the research [42], a higher signal variation was obtained by artificial defects situated closer to the surface (probe). A simulation [43] showed that the signal variation of the notch-type defect had one peak, while that of a bigger spherical defect (3 mm diameter) caused a double peak, which confirms the results of this experiment.

## 5. Conclusions

In this experiment, the eddy current testing method was used to inspect SS 316L stainless steel samples created with selective laser melting (SLM) technology. Artificial defects, designed in various shapes and sizes, caused partial impedance elements (reactance and resistance) variations, which consequently influenced the shape of the curves on a device display within both IMPEDANCE and SWEEP regimes. Based on the obtained data, a few conclusions can be derived:

- Various reactance and resistance values, with various follow-up signal deviations according to the different shapes and sizes of the designed artificial defects in the article, prove that the eddy current non-destructive testing method has all the prerequisites for detecting defects in additively manufactured parts made of used material.
- For the experimental setup of the parameters of a measurement device, it is important to know if the evaluated defect reaches the surface of the material or if it is included in a subsurface layer. Same as in the case of the common eddy current testing of materials, a higher frequency and consequently a higher sensitivity can be set for the first mentioned, since the skin effect significantly affects the eddy current measurement.
- Additionally, the selection of the available identification regimes (IMPEDANCE or SWEEP) is important, according to the defect kind. For the inner defects in the subsurface layer, the better choice is unequivocally the SWEEP mode because of the significantly higher noise and lift-off effect of the IMPEDANCE regime signal during the use of higher frequencies and GAIN values. On the other side, the IMPEDANCE mode is better for surface reaching defects because of its better depiction of the obtained data. For both regimes, it is positive to differ the setup according to the size of the defect, where a 1 mm diameter or depth (for the grooves) could be a boundary due to the high signal difference of the smaller and larger defects.
- As can be seen for samples S3 and S5, cavities with a 0.2 mm diameter were not printed, or were just partially printed. So, it is important to take the possibilities of printing technology into account. However, it can be stated that the smallest defect that can be detected has a 0.5 mm diameter and depth for the groove type. The highest reached depth of a place of defect can be 6.5 mm.
- Regarding the defect shape influence, for the groove type shape, a higher Vmax deviation and a stronger peak in the vertical direction was obtained, which applies to both regimes. In the case of the circular and spherical shapes, the Vmax and Hmax variations were not so different. For the SWP mode, double peaks were obtained and for the IMP mode, a wave-resembling curve was obtained. In both cases, the size of the peak depended on the size and depth of the defect.
- Another factor, which is a follow-up to a previous point, is the presence of powder in a cavity. If a defect is filled with powder, the signal is not influenced with such a magnitude as an empty one is. This is due to the smaller sensitivity of the eddy currents on a medium with some density compared to ones with empty space.

According to the obtained data and summarized conclusions, some issues are still present and require other follow-up studies. Considering this fact, the standard penetration depth was calculated based on the conductivity measurement and the reached penetration depth was probably higher; thus, this phenomenon deserves more comprehensive and detailed research. Regarding, that measurements were done with hand-probe, it is on the place to consider the design of jig, for restriction of lift-off effect. Since the method shows potential for AM stainless steel material, it is clearly necessary to conduct a similar experiment on samples of different shapes and other AM materials, such as aluminum or titanium alloys. In comparison with X-ray technology, the eddy current method is cheaper, simpler and non-harmful to health. Additionally, the eddy current method is more suitable for production conditions. In comparison with ultrasonic testing, the eddy current method is more sensitive to surface and near-surface defects, whereas ultrasonic testing is commonly used for bulk defects. Combining the UT and ET methods, the comprehensive (both surface and bulk defects) material identification of additive manufacturing products can be reached.

## Figures and Tables

**Table 1:** SS 316L material composition [46].

Element	Fe	Cr	Ni	Mo	Mn	Si	N	O	P	C	S
**Mass (%)**	Balance	16–18	10–14	2–3	≤2	≤1	≤0.1	≤0.1	≤0.045	≤0.03	≤0.03

**Table 2:** Calculated standard penetration depth values.

Frequency *f* [Hz]	Standard Penetration Depth *δ* [mm]	
	AISI 316L	SS 316L
500	19.36	16.03
1000	13.69	11.33
2000	9.68	8.01
3000	7.9	6.54
4000	6.84	5.67
5000	6.12	5.07
6000	5.59	4.63
7000	5.17	4.28
8000	4.84	4.01
9000	4.56	3.78
10,000	4.33	3.58
20,000	3.06	2.53
30,000	2.5	2.07
40,000	2.16	1.79
50,000	1.94	1.6
60,000	1.77	1.46
70,000	1.64	1.35
80,000	1.53	1.27
90,000	1.44	1.19
100,000	1.37	1.13

**Table 3:** Eddy current testing conditions settings.

Sample	Defects	*f* [Hz]	Hgain [dB]	Vgain [dB]	Angle [°]	Depth [mm]	DSP MODE
**S1**	D1, D2, D3, D4	30,000	42.4	64	225	2.07	IMP
**S2**	D2, D3	90,000	64.5	63.5	134	1.19	IMP
	D4, D5, D6	5000	71	71	161	5.07	IMP
**S3**	D2, D3	15,000	65	74	145	2.93	SWP
	D4, D5, D6	10,000	65	74	135; 85(D4)	3.58	SWP
**S3 (reversed)**	D5, D6	10,000	63	58.5	180	3.58	IMP
**S4**	D1, D2, D3, D4	15,000	65	76	115	2.93	SWP
**S5**	D2, D3, D4	50,000	66	77	40	1.6	SWP
	D5, D6	15,000	65	75	90	2.93	SWP
**S6**	D1, D2, D3, D4	25,000	66	77	105	2.27	SWP

**Table 4:** Vmax and Hmax signal-to-defect variations.

Sample	Vmax						Hmax					
	D1	D2	D3	D4	D5	D6	D1	D2	D3	D4	D5	D6
**S1**	−0.1	−0.6	−1.5	−3.0	-	-	0	0	0	0	-	-
**S2**	-	0.8	2.8	0.4	1.3	4.4	-	0.4	1.3	0.1	0.4	2.0
**S3**	-	0.8	0.9	−1.1/0.6	0.7	2.2	-	0.1	0.1	0.3/0.5	0.3	1.5
**S4**	0.7	−0.4	0.6	0.4	-	-	0.3	0.1	−0.1	−0.1	-	-
**S5**	-	0.3	0.4	0.6	−0.7	2.9	-	−0.4	−0.9	0.6	0.5	1.3
**S6**	−1.3	−0.7	−1.1	0.9	-	-	0.3	0.1	0.2	−0.2	-	-

## Data Availability

Not applicable.
